# Integrating Full-Length and Second-Generation Transcriptomes to Elucidate the ApNPV-Induced Transcriptional Reprogramming in *Antheraea pernyi* Midgut

**DOI:** 10.3390/insects16080792

**Published:** 2025-07-31

**Authors:** Xinlei Liu, Ying Li, Xinfeng Yang, Xuwei Zhu, Fangang Meng, Yaoting Zhang, Jianping Duan

**Affiliations:** 1 Henan Key Laboratory of Funiu Mountain Insect Biology, China-UK International Joint Laboratory for Insect Biology of Henan Province, Nanyang Normal University, Nanyang 473061, China; 20540140251@nynu.edu.cn (X.L.); lying06070501@163.com (Y.L.); 2 Laboratory of Tussah Genetics and Breeding, Henan Institute of Sericulture Science, Zhengzhou 450008, China; 13193685292@163.com (X.Y.); hnsckyzxw@126.com (X.Z.); 15036230928@163.com (F.M.); ckyyaoting@126.com (Y.Z.)

**Keywords:** *Antheraea pernyi*, transcriptome, alternative splicing, immunity, energy metabolism

## Abstract

The midgut of *Antheraea pernyi* plays a critical role in antiviral defense. However, its transcriptional complexity remains poorly understood. Here, a full-length (FL) transcriptome atlas of *A. pernyi* midgut was developed by integrating PacBio Iso-Seq and RNA-seq techniques. The transcriptome sequences included 1850 novel protein-coding genes, 17,736 novel alternative isoforms, 1664 novel long non-coding RNAs (lncRNAs), and 858 transcription factors (TFs). In addition, 2471 alternative splicing (AS) events and 3070 alternative polyadenylation (APA) sites were identified. Moreover, 3426 and 4796 differentially expressed genes (DEGs) and isoforms were identified after ApNPV infection, respectively, besides the differentially expressed lncRNAs (164), TFs (171), and novel isoforms of *ApRelish* (1) and *ApSOCS2* (4). Enrichment analyses showed that KEGG pathways related to metabolism were suppressed, whereas GO terms related to DNA synthesis and replication were induced. Furthermore, the autophagy and apoptosis pathways were significantly enriched among the upregulated genes. Protein–protein interaction network (PPI) analysis revealed the coordinated downregulation of genes involved in mitochondrial ribosomes, V-type and F-type ATPases, and oxidative phosphorylation, indicating the disruption of host energy metabolism and organelle acidification. Moreover, coordinated upregulation of genes associated with cytoplasmic ribosomes was observed, suggesting that the infection by ApNPV interferes with host translational machinery. These results show that ApNPV infection reprograms energy metabolism, biosynthetic processes, and immune response in *A. pernyi* midgut. Our study provides a foundation for elucidating the mechanisms of *A. pernyi*–virus interactions, particularly how the viruses affect host defense strategies.

## Simple Summary

The midgut of *Antheraea pernyi* serves as a primary defense barrier against *A. pernyi* nuclear polyhedrosis virus (ApNPV); however, the molecular interplay between the virus and host defense remains incompletely characterized. In this investigation, PacBio Iso-Seq and RNA-seq data were integrated for comprehensive profiling of the midgut transcriptome and dynamic responses to orally infected viruses that had formed polyhedra in the hemolymph. Many metabolism-related genes were downregulated. However, genes related to *A. pernyi* cytoplasmic translation, autophagy, and apoptosis were found to be upregulated. In addition, several differentially expressed long non-coding RNAs and transcription factors were identified. These results provide initial insights into how ApNPV affects host resources to facilitate viral replication and midgut escape.

## 1. Introduction

*Antheraea pernyi* (Guérin-Méneville, 1855), a lepidopteran economic insect, has been artificially reared in China for centuries, prized for its high-quality silk and applications in traditional medicine [1]. *A. pernyi* offers new insights into lepidopteran developmental biology; however, its susceptibility to pathogens in outdoor farming environments poses major challenges to productivity [2]. The midgut, a multifunctional tissue central to immune defense and nutrient absorption, serves as the primary interface against pathogenic invasion [3,4]. Upon ingestion, pathogens trigger a cascade of immune mechanisms, including physical barriers, antimicrobial peptide (AMP) secretion, and pattern recognition receptor (PRR)-mediated signaling pathways [4,5,6]. The key molecular players in these processes include peptidoglycan recognition proteins (PGRPs) [7,8], which recognize pathogen-associated molecular patterns (PAMPs), and serine protease cascades [9,10,11,12], which activate prophenol oxidases (PPOs) to encapsulate pathogens through melanization. These pathways intersect with conserved immune signaling pathways, such as the Toll [13,14]/immune deficiency (IMD) [15] and Janus kinase-signal transducer and activator of transcription (JAK/STAT) pathways [16], which regulate AMP expression and antiviral responses, respectively. Despite advances in the characterization of these mechanisms, critical gaps persist, including the molecular details of antiviral tolerance and the role of immune suppressors in maintaining homeostasis.

Viral replication and amplification depend entirely on the host cell’s resources. Once inside the host, the virus disrupts the normal regulation of host gene expression, suppresses the host immune response, and redirects host cells to synthesize the required proteins and nucleic acids [17]. In the late stages of infection, the virus achieves its own dissemination by exploiting cellular autophagy and apoptosis pathways [18]. Studies on *Bombyx mori* have shown that silkworms employ multiple strategies to inhibit viral replication and spread, including the NF-κB pathway and RNA interference [16]. However, the virus has evolved several mechanisms to evade host immunity, such as induction of BmSerpin2 to suppress host melanization [19] and hijacking cellular transcription machinery [20]. The precise mechanisms by which ApNPV reprograms *A. pernyi* cellular machinery remain unclear.

In previous studies on the *A. pernyi* midgut, particularly those analyzing immune responses to ApNPV infection, only short-read sequencing was performed [21,22], which limited the ability to identify splice variants and non-coding elements. Third-generation sequencing techniques, especially PacBio single-molecule in real-time (SMRT) sequencing, allow FL transcriptome profiling and solve the problems associated with short reads [23]. AS is an important mechanism of gene expression regulation that generates various mRNA isoforms, thereby influencing the functional diversity of proteins [24]. In wheat, AS of the *Triticum aestivum* histidine-rich calcium-binding protein gene is associated with resistance to *Fusarium* head blight (FHB), which determines susceptibility or resistance to FHB by regulating pre-mRNA processing [25]. Similarly, in *Arabidopsis*, AS acts as a key modulator of the immune response and plays a critical role in coping with biotic stress [26]. In *B. mori*, two AS forms of *PGRP2* show different functions in response to pathogens [27]. Further research on AS is required to elucidate the potential gene regulatory mechanisms in *A. pernyi*. LncRNAs are involved in various biological processes. In *B. mori*, the lncRNA Bmdsx-AS1 participates in the AS of the sex-determination gene *Bmdsx* [28]. Moreover, profiling of lncRNA expression in *B. mori* larvae following infection with BmNPV [29,30] and BmCPV [31] revealed that lncRNAs are involved in antiviral infection and host immune responses. However, studies on AS and lncRNAs in *A. pernyi* are still in their infancy.

Here, we aimed to elucidate a comprehensive FL transcriptome atlas of the *A. pernyi* midgut. PacBio SMRT and RNA-seq approaches were integrated, which resulted in improved annotations of some reference loci active in *A. pernyi* midgut and identification of many novel genes, lncRNAs, and TFs. Additionally, we discovered that AS is an important regulatory pathway for genes associated with innate immunity in *A. pernyi*. Moreover, energy metabolism and cytoplasmic ribosomes are dysregulated following ApNPV infection. Our results not only address critical gaps in midgut genomic annotation but also pave the way for elucidating the molecular mechanisms underlying the dual roles of genes expressed in the midgut and pathogen–host interactions.

## 2. Materials and Methods

### 2.1. Preparation of Samples

The univoltine strain of *A. pernyi* was reared on oak leaves at the *A. pernyi* base of the Henan Sericulture Research Institute in China. The uninfected midgut tissues of three fifth-instar larvae were dissected and isolated. Total RNA was extracted from the collected tissue samples using TRIzol reagent (Invitrogen, Carlsbad, CA, USA), according to the manufacturer’s protocol. The extracted RNA was purified using RNeasy Mini Kits (Qiagen, Valencia, CA, USA) to remove contaminants and improve quality. The integrity, purity, and concentration of the RNA samples were assessed using a NanoDrop spectrophotometer (Thermo Fisher Scientific, Waltham, MA, USA), Agilent 2100 Bioanalyzer (Agilent, Santa Clara, CA, USA), and agarose gel electrophoresis. High-quality RNA samples were stored at −80 °C for subsequent sequencing and other downstream applications.

### 2.2. PacBio Sequencing

For cDNA library construction, high-quality RNA was reverse-transcribed using a Clontech SMARTer^TM^ PCR cDNA Synthesis Kit (Clontech, San Jose, CA, USA). Magnetic beads (Vazyme, Nanjing, China) were used to purify cDNA and remove fragments smaller than 1 kb in length. After end-repair, SMRT dumbbell adapters were ligated, and unligated adapters were degraded by exonuclease treatment, followed by purification. The final library was quantified using a Qubit 3.0 fluorometer (Thermo Fisher Scientific, USA). The fragment size distribution was assessed using an Agilent 2100 bioanalyzer to ensure quality prior to sequencing.

The cDNA library was sequenced using the PacBio platform (Pacific Biosciences, Menlo Park, CA, USA), and polymerase reads were processed using SMRT Link software v10.1 (Pacific Biosciences, USA). Adapter sequences were removed to generate subreads, which underwent error correction to produce highly accurate circular consensus sequences (CCSs). CCS reads were analyzed for the presence of 5′ primers, 3′ primers, and poly(A) tails and classified as FL sequences, full-length non-chimeric sequences (FLNCs), or FLNCs with poly(A) tails. Only sequences containing both 5′ and 3′ primers along with a poly(A) tail were considered to be FL transcripts.

### 2.3. Data Processing and Gene Model Optimization

FL transcripts were mapped to the reference genome using GMAP [32,33] with a set of predefined locus classification criteria. A transcript was assigned to a known locus if it demonstrated sufficient alignment overlap (≥20% in the same orientation) with an annotated transcript [34], displayed a unique splicing pattern if it was a single-exon transcript differing from reference annotation, or contained a novel splice site. The longest transcript was retained when multiple transcripts shared the same splice sites. Redundancy among transcripts was minimized by applying one of the following two key filtering criteria: a high global pairwise identity (PID) threshold or confirmation through support from multiple FLNC reads for transcripts with a slightly low PID.

FL status was determined based on alignment with reference transcripts, ensuring a minimal overlap in the same orientation and matching at the first splice donor site. Transcripts were designated as novel genes if they exhibited minimal overlap or were oriented in an opposite direction relative to the known annotations. Similarly, novel isoforms were identified based on the presence of unannotated splice junctions or structural differences in single-exon transcripts. To further optimize the gene models, additional strategies were incorporated into our analysis, which involved isoform identification of transcripts at the same loci, removal of redundant transcripts, and filtering of low-confidence transcripts to detect and correct potential misannotations. Here, the low-confidence transcripts represented those with PID below 99% and only one FLNC support. These procedures allowed us to refine gene boundaries and update exon–intron definitions, thereby improving the overall quality and accuracy of the gene annotations.

### 2.4. Functional Annotation of Genes and Identification of lncRNAs

Diamond v2.0.7 [35] was used to perform functional annotation of the novel genes based on the NCBI non-redundant (NR) protein sequence database, followed by the integration of data from Gene Ontology (GO) [36], Swiss-Prot [37], and Cluster of Orthologous Groups of proteins (COG/KOG) [38]. Additionally, KOBAS [39] was used to determine the potential functions of these genes using the Kyoto Encyclopedia of Genes and Genomes (KEGG) [40]. To further identify lncRNAs, all FL transcripts that failed to align with the aforementioned databases were analyzed for coding potential using CPC2 [41], CNCI [42], CPAT [43], and PLEK [44] bioinformatic tools.

### 2.5. Identification of APA and AS Events

In eukaryotes, pre-mRNAs are generally processed via AS, which gives rise to multiple mRNA isoforms using different combinations of splice sites [45]. In addition to AS, APA is a critical post-transcriptional regulatory mechanism that affects transcript stability, localization, and translation efficiency [46]. AS generates mRNA diversity by varying splice-site usage, whereas APA modulates transcript stability, localization, and translation. We classified and quantified AS events based on the FL isoforms using Astalavista [47] and detected all gene-associated poly(A) sites and their corresponding isoform counts using Tapis [48].

### 2.6. TF Identification and Analysis

TF annotation in *A. pernyi* was performed using HMMER [49] based on the 73 hidden Markov model profiles of TF families retrieved from the AnimalTFDB3.0 database (https://guolab.wchscu.cn/AnimalTFDB#!/, accessed on 17 February 2025). Candidate TF genes were functionally classified into families based on their domain architecture and sequence homology. This integrative approach enabled the systematic characterization of TFs that govern the immune and metabolic processes in the midgut of *A. pernyi*.

### 2.7. Comparative Transcriptome Analysis of DEGs After ApNPV Infection

RNA-seq data for the infected midgut with ApNPV [50] were downloaded and reanalyzed based on the *A. pernyi* genome [32] and the improved gene annotations above. Importantly, the sequencing samples in the 2016 study [50] were dissected from the infected *A. pernyi* larvae after polyhedral formation was observed in the hemolymph. Differential expression analysis was performed using DESeq2 [51] [|log2(FoldChange)| > 1 and *p*-value adjusted < 0.05]. Enrichment analysis was conducted with the ClusterProfile v4.16.0 (https://www.bioconductor.org/packages/release/bioc/html/clusterProfiler.html, accessed on 29 July 2025). Qvalue was used to justify the enrichment significance. Diamond v2.0.7 [35] was used to map significant DEGs to the STRING database (https://cn.string-db.org/, accessed on 23 February 2025) using default parameters, and *B. mori* was used as the closest match. The MCODE plugin in Cytoscape v3.10.3 (https://apps.cytoscape.org/apps/mcode, accessed on 29 July 2025) was used to identify high-density protein interaction modules under default parameters (node score cutoff = 2, sensitivity = 0.2, cluster score ≥ 2). The cluster score, which reflects the topological density of a module, was used to prioritize functionally relevant genes. Each detected module likely comprises genes that engage in direct or indirect interactions, potentially participating in shared biological processes. Two highest-density modules were selected for downstream functional analysis and visualization of some functionally similar blocks.

## 3. Results

### 3.1. PacBio Data Output

**Figure 1:**
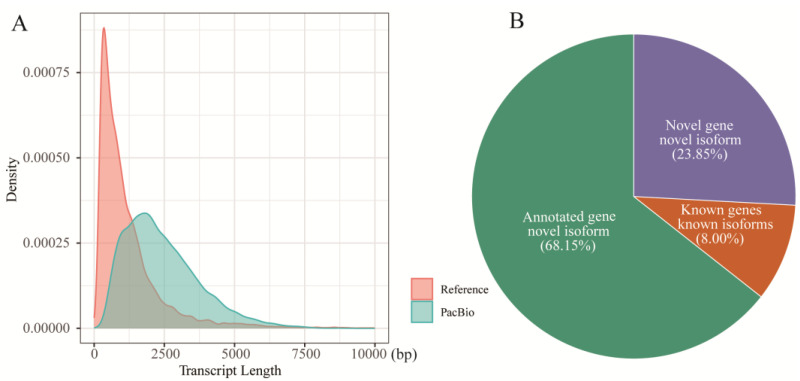
PacBio data from the FL transcriptome of the uninfected *A. pernyi* midgut. (**A**) Comparison of the transcript length between reference and PacBio annotations. (**B**) Details of effective FL isoforms mapped to the *A. pernyi* genome.

Accurate gene annotation is critical for investigating midgut immune functions. To explore the complex immune responses in the midgut of *A. pernyi* following ApNPV infection, we generated full-length transcriptome data for uninfected tissue. Using PacBio long-read sequencing, we generated 39.48 Gbp of raw data, yielding 550,771 raw reads (Table 1). After adapter trimming, 19,093,122 subreads were retained. Filtering of these subreads produced 378,156 high-quality CCS reads. Next, we selected FLNC reads containing intact poly(A) tails, yielding 288,759 FLNC reads (0.55 Gb; mean length, 1888 bp; N50 = 2113 bp). The FLNC reads were mapped to the reference genome using GMAP, resulting in 248,345 raw alignments. After rigorous removal of redundant transcripts and filtering of low-confidence sequences, 19,279 non-redundant transcript isoforms were finalized. Notably, these PacBio-derived isoforms were significantly longer than the reference transcripts (Figure 1A).

### 3.2. Determination of Novel Genes, LncRNAs, and TFs

**Figure 2:**
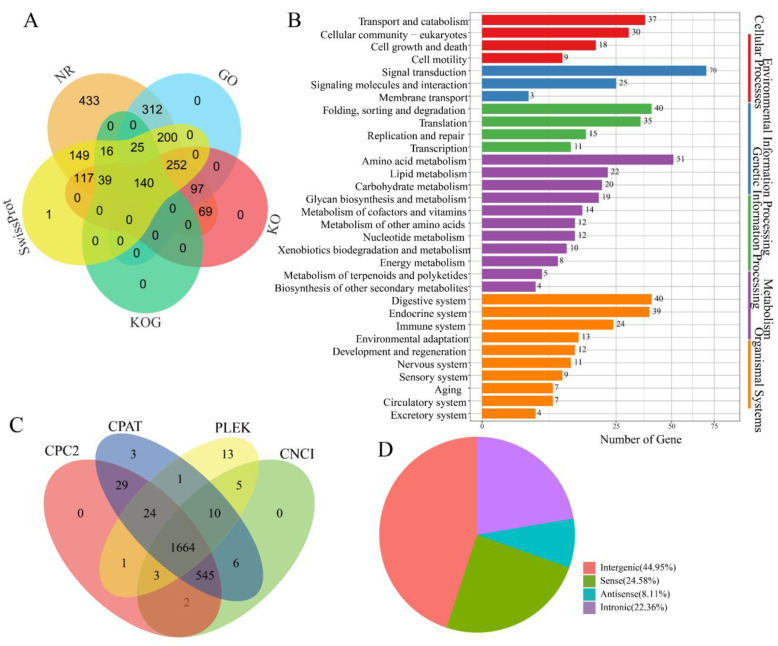
Determination of the genes and lncRNAs expressed in the midgut of uninfected *A. pernyi*. (**A**) Functional annotation of novel protein-coding genes using databases NR, KOG, GO, KEGG, and Swiss-Prot. (**B**) Novel protein-coding genes categorized according to the KEGG annotations. (**C**) Assessment of the lncRNAs in the midgut using CPC2, CPAT, PLEK, and CNCI bioinformatic tools. (**D**) Classification of lncRNAs.

To identify novel isoforms, 19,279 high-confidence and non-redundant transcript isoforms were compared with the *A. pernyi* genome. Among these isoforms, 13,138 (68.15%) were identified as novel isoforms of known genes (Figure 1B), 4598 (23.85%) were designated as novel isoforms derived from newly identified genes, and 1543 (8%) were observed to be the known isoforms of known genes. Subsequently, the newly identified genes and novel isoforms were functionally annotated. A total of 1850 novel genes were successfully annotated using public databases, including GO, KOG, KEGG, NR, and Swiss-Prot (Figure 2A and Table S1). Among these, 1026 novel protein-coding genes were annotated in the GO database, and 368 novel genes were annotated using KEGG annotations. GO annotation categorized these genes into 35 distinct terms, including 13 molecular functions, 20 biological processes, and 2 cellular components (Figure S1). KEGG annotation categorized the genes into 214 pathways and grouped them into five major functional categories: cellular processes, environmental information processing, genetic information processing, metabolism, and organismal systems (Figure 2B). In particular, some potentially immune-related genes were identified for the first time in *A. pernyi*. For example, the novel gene GWHABGR00000010.119 was annotated in both the GO and KEGG databases and was associated with GO:0042981 (regulation of apoptosis), GO:0007165 (signal transduction), and K02373 [Fas associated via death domain (FADD)]. The gene was mapped to nine KEGG pathways and designated via Swiss-Prot analysis as a homolog of the *Drosophila melanogaster* FADD protein (sp|Q9V3B4|FADD_DROME) with conserved apoptotic domains. Similarly, the novel gene GWHABGR00000001.115 was annotated with the GO terms zinc ion binding (GO:0008270), integral component of the membrane (GO:0016021), peptidoglycan catabolic process (GO:0009253), innate immune response (GO:0045087), and N-acetylmuramoyl-L-alanine amidase activity (GO:0008745). It was assigned to KO entry K01446 (PGRP) and mapped to the Toll and IMD signaling pathways (ko04624). Swiss-Prot analysis identified it as a homolog of the *Trichoplusia ni* peptidoglycan recognition protein (sp|O76537|PGRP_TRINI). In addition, some immune-related genes, such as *SOCS2*, *Relish*, *Uev1A*, and *SOS*, were first discovered to contain more than one isoform in *A. pernyi*.

LncRNAs have been widely reported to play crucial roles in various biological processes, including cell differentiation, epigenetic regulation, and transcriptional control [52,53]. In *B*. *mori*, lncRNAs have been shown to modulate immune responses [29]; however, their roles in *A. pernyi*, particularly in the midgut, remain largely unknown. Unannotated isoforms were used to identify the potential lncRNAs. In total, 1664 lncRNAs were identified in the *A. pernyi* midgut (Figure 2C). Among these, 748 lncRNAs (44.95%) were classified as intergenic, whereas 409 (24.58%) and 135 (8.11%) were annotated as sense and antisense lncRNAs, respectively. In addition, 372 intronic lncRNAs, accounting for 22.36% of the total, were identified (Figure 2D). Some lncRNAs may be involved in the immune and metabolic functions in the midgut of *A. pernyi* [54].

TFs serve as the primary architects of gene expression, orchestrating the molecular mechanisms underlying insect development, environmental adaptation, and disease resistance [55,56]. In total, 858 TFs belonging to 56 families were identified in the midgut of *A. pernyi* (Table S2). Of these, 346 TFs (40.3%) belong to the zf-C2H2 family, followed by the homeobox family with 68 members (7.9%), BTB family with 66 members (7.7%), bHLH family with 50 members (5.8%), and the HTH family with 38 members (4.4%). In addition, the THAP family comprised 29 members (3.4%), the Retinoid_X_Receptor-like family had 26 members (3%), the HMG family had 24 members (2.8%), and the bZIP family had 23 members (2.7%). The Forkhead and MYB families each included 21 members (2.4%), the zf-CCCH family included 15 members (1.7%), and the T-box family included 10 members (1.2%). Moreover, the ETS, PAX, and zf-BED families each comprised eight members (0.9%), the GATA family had seven members (0.8%), and the zf-LITAF-like family had six members (0.7%). The ARID and MH1 families each had five members (0.6%), whereas the CSD, E2F, MBD, NFYB, and NFYC families each had four members (0.5%). Seven families, including CSL, CUT, DM, Pou, RFX, RHD, and Runt, each contained three members (0.4%). Nine families, including GCFC, LRRFIP, Nrf1, SRF-TF, TSC22, Tub, zf-C2HC, zf-MIZ, and zf-NF-X1, each contained two members (0.2%). Finally, 15 families, including AP-2, CBF, CP2, CSRNP_N, DACH, HMGA, HPD, HSF, NCU-G1, NF-Y, P53, PC4, STAT, TEA, and COE, were represented by a single member (0.1%).

### 3.3. Complex Regulation of RNA Transcription by AS and APA

**Figure 3:**
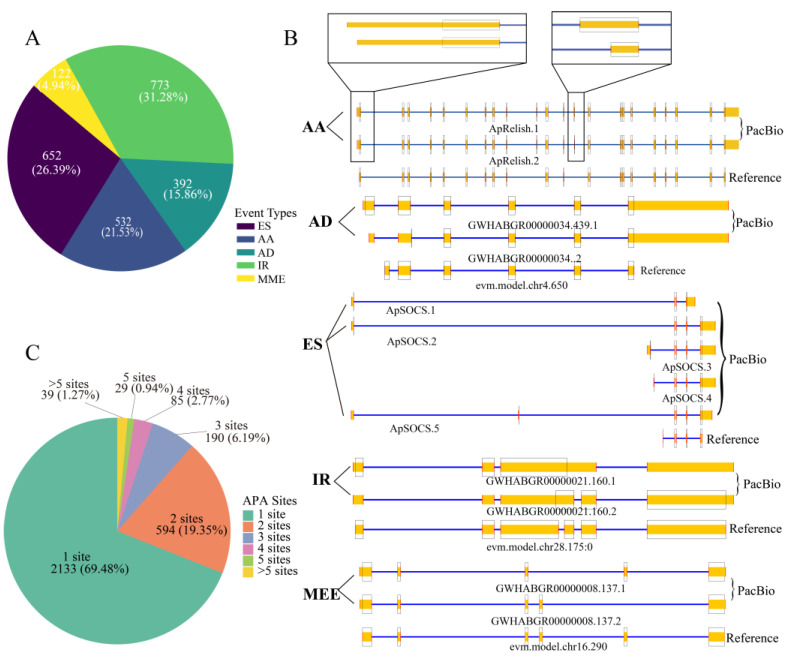
AS and APA events detected in the midgut of uninfected *A. pernyi*. (**A**) Frequency distribution of AS events in the midgut. (**B**) Representative AS patterns. PacBio FL isoforms (top) are aligned with reference genome annotations (bottom). The coding sequence (CDS) is demarcated by a black box. Genes on the negative and positive strands are annotated using blue and yellow rectangles, respectively. (**C**) Gene counts are classified by distinct APA event types in FL data.

AS is a fundamental mechanism that enhances the complexity of gene expression and plays crucial roles in cell differentiation and organismal development [57,58]. A total of 2471 AS events were first identified in the midgut after analysis of the FL transcript isoforms (Figure 3A and Table S3). These included 652 exon-skipping (ES), 532 alternative acceptor (AA), 392 alternative donor (AD), 773 intron retention (IR), and 122 mutually exclusive exon (MEE) events. There were 1250 genes (5.39% of the total annotated genes) discovered using AS to regulate their expression in the midgut. Notably, a few immune-related genes use AS to regulate their expression. For example, *ApRelish* generates two splice variants mediated by AA splicing mechanisms, whereas *ApSOCS2* produces three isoforms (ApSOCS2.1, ApSOCS2.2, and ApSOCS2.5) arising from ES splicing events (Figure 3B).

APA is a widespread RNA processing mechanism present in all eukaryotic species and is considered a major mode of regulating of gene expression [59]. Analysis of the FL data revealed that 2133 genes (69.48%) harbored a single APA site, 594 genes (19.35%) contained two APA sites, 190 genes (6.19%) exhibited three APA sites, 85 genes (2.77%) had four APA sites, 29 genes (0.94%) had four APA sites, and 39 genes (1.27%) possessed more than five APA sites (Figure 3C and Table S4). For example, the Toll-interacting protein (Tollip) gene (evm.TU.chr6.235), located on the positive strand and supported by 58 aligned FLNC reads, was identified to contain three distinct APA sites. The three APA sites of the Tollip gene may be involved in translation regulation, but the underlying mechanisms remain to be clarified.

### 3.4. Transcriptional Reprogramming in A. pernyi Midgut Following ApNPV Infection

**Figure 4:**
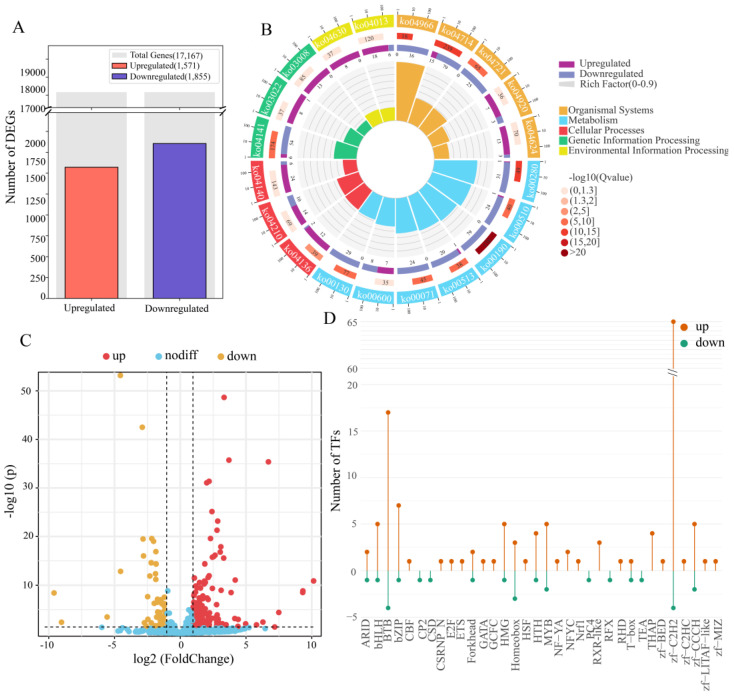
Composite overview of transcriptomic and regulatory responses in *A. pernyi* midgut following ApNPV infection. (**A**). Bar chart showing the count of genes significantly upregulated versus downregulated gene numbers. (**B**). Circular KEGG pathway enrichment plot of the top enriched pathways among the DEGs, with node size proportional to gene ratio and node color intensity. Qvalue is the *p* value adjusted for multiple hypothesis testing. Rich factor is the ratio of DEGs enriched in a given pathway to the total genes annotated to that pathway. Background auxiliary lines: 1 grid = 0.1 ich factor. (**C**). Volcano plot showing differential expression of lncRNA. (**D**). Lollipop chart showing the number of upregulated (red) and downregulated (blue) genes in each TF family following ApNPV induction. Family names are listed on the *x*-axis.

To more accurately define the gene expression features induced by ApNPV and surmount the constraints imposed by second-generation sequencing and de novo assembly in 2016, comparative transcriptome analysis with reference using FL-improved annotations and RNA-seq data [50] was conducted. The results revealed 1571 and 1855 genes upregulated and downregulated, respectively, after ApNPV infection (Figure 4A), different from the 2183 upregulated and 2989 downregulated genes discovered in 2016 [50]. The expression patterns of some genes and transcripts involved in classical immune pathways changed (Figure S2 and Table S5). For example, four transcripts were differentially expressed in the Toll pathway. Toll1.1, Toll2.1, and PSH expression were significantly upregulated, whereas MyD88 expression was downregulated. Eight transcripts were differentially expressed in the IMD pathway. The Pirk variants, Dredd, Tab2.1, Tab2.2, ApRelish.1, and ApRelish.2, were markedly upregulated, whereas the two Uev1A isoforms were downregulated. Nine transcripts were differentially expressed in the JAK/STAT pathway. STAM.1, STAM.2, two SOS transcripts, PI3K, CBP/P300, ApSOCS2.2, and ApSOCS2.3 were upregulated, whereas SHP2 was significantly downregulated. However, the expression of novel FADD and PGRP genes exhibited no significant difference in the infected *A. pernyi* midgut, similar to the expression pattern of *ApPGRP-LE* after viral infection [8].

GO and KEGG enrichment analyses were conducted to explore the important functions of DEGs related to the stress response after ApNPV infection. In total, 2224 DEGs, including 881 upregulated and 1343 downregulated genes, were enriched in GO terms. The top four GO terms enriched by the upregulated genes (Table S6) were as follows: 129 upregulated genes were enriched in catalytic activity, acting on DNA (GO:0140097), 115 in nucleotidyltransferase activity (GO:0016779), 109 in DNA polymerase activity (GO:0034061), and 159 in catalytic activity, acting on nucleic acids (GO:0140640). In addition, 107 genes were enriched for RNA-directed DNA polymerase activity (GO:0003964), 153 for transferase activity and transfer of phosphorus-containing groups (GO:0016772), and 219 for transferase activity (GO:0016740). Regarding cellular components, 22 upregulated genes were enriched in the nucleolus (GO:0005730), 48 were enriched in acyltransferase activity (GO:0016746), and 191 were enriched in the nucleus (GO:0005634). In contrast, the top 10 GO terms enriched by the downregulated genes were primarily related to cellular components and molecular functions. Specifically, 149 downregulated genes were enriched in the organelle membrane (GO:0031090), 309 in the cytoplasm (GO:0005737), and 730 in catalytic activity (GO:0003824). Moreover, 102 genes were enriched in mitochondria (GO:0005739), 65 in the mitochondrial membrane (GO:0031966), and 66 in the mitochondrial envelope (GO:0005740). Additionally, 71 genes were enriched in the organelle envelope (GO:0031967), 71 in the envelope (GO:0031975), and 72 in the membrane protein complex (GO:0098796). Regarding molecular functions, 192 downregulated genes were enriched in oxidoreductase activity (GO:0016491). Compared to the 2016 study [50], some GO terms related to DNA synthesis and transcription (GO:0003676, GO:0005634, GO:0032774, etc.) and metabolism (GO:0016491, GO:0055114, GO:0003824, etc.) were also enriched, but some important GO terms, for example, the catalytic activity, acting on DNA (GO:0140097) and nucleic acid (GO:0140640), and membrane protein complex (GO:0098796), were only significantly enriched here, indicating the improved role of accurate gene annotations in enrichment analysis.

A total of 1194 DEGs, including 362 upregulated and 832 downregulated genes, were enriched for 202 and 234 KEGG pathways, respectively. The significantly enriched pathways provided important insights into the distinct biological processes associated with changes in gene expression (Figure 4B and Table S7). The top 10 upregulated pathways included autophagy (ko04136 and ko04140), apoptosis (ko04210), Toll/IMD signaling (ko04624), and JAK/STAT signaling (ko04630) pathways. Conversely, the 10 most downregulated pathways were primarily associated with metabolic, organismal system, and genetic information processing functions. These included oxidative phosphorylation (ko00190), valine, leucine, and isoleucine degradation (ko00280), thermogenesis (ko04714), collecting duct acid secretion (ko04966), N-glycan biosynthesis (ko00510 and ko00513), fatty acid degradation (ko00071), protein processing in the endoplasmic reticulum (ko04141), ubiquinone and other terpenoid–quinone biosynthesis (ko00130), and the synaptic vesicle cycle (ko04721). Compared with the 2016 results [50], some pathways associated with basal transcription factors (ko03022), DNA replication (ko03030), spliceosome (ko03040), biosynthesis of amino acids (ko01230), and oxidative phosphorylation (ko00190) were also enriched. However, other pathways, especially autophagy–other (ko04624), Toll and Imd signaling (ko04920), adipocytokine signaling (ko04136), thermogenesis (ko04966), collecting duct acid secretion (ko04721), and synaptic vesicle cycle (ko04714), were only significantly enriched here, improving the insight into the changes in the important pathways during ApNPV invasion.

The changes in the expression levels of lncRNAs and TFs in response to ApNPV infection were also examined. In total, 164 differentially expressed lncRNAs were identified, of which 120 were upregulated and 44 were downregulated (Figure 4C and Table S8). These differentially expressed lncRNAs may play a role in immunity or metabolism in *A. pernyi* midgut [54] and require further investigation. In comparison, 171 TFs exhibited differential expression in the *A. pernyi* midgut after ApNPV infection, of which 144 were upregulated, and 27 were downregulated (Figure 4D and Table S9). Among these TF families, the zf-C2H2 family showed the highest number of differentially expressed TFs, with sixty-five upregulated and four downregulated TFs. The BTB, bZIP, MYB, bHLH, and HMG families included seventeen, seven, five, five, and five upregulated and four, one, two, one, and one downregulated members, respectively. Fifteen families featured only upregulated transcription factors. Among these, the THAP family had the highest number of upregulated members (4). The RXR_like and NFYC families contained three and two upregulated members, respectively. The HSF, Nrf1, and ETS families each contained one upregulated member. In contrast, the CP2, CSD, PC4, RFX, and TEA families each contained one downregulated member. The only exception was the homeobox family, which included three upregulated and three downregulated members. In *B. mori*, BmNPV enhanced infection by downregulating the transcription factor *BmFoxO*, whereas overexpression of *BmFoxO* strengthened host antiviral resistance [60]. Similarly, *STAT* was closely related to the *B. mori* innate immune response [61]. The differentially expressed TFs identified in this study may have potential immune functions; however, their specific regulatory mechanisms need to be investigated further.

### 3.5. Construction of the PPI Network of DEGs

**Figure 5:**
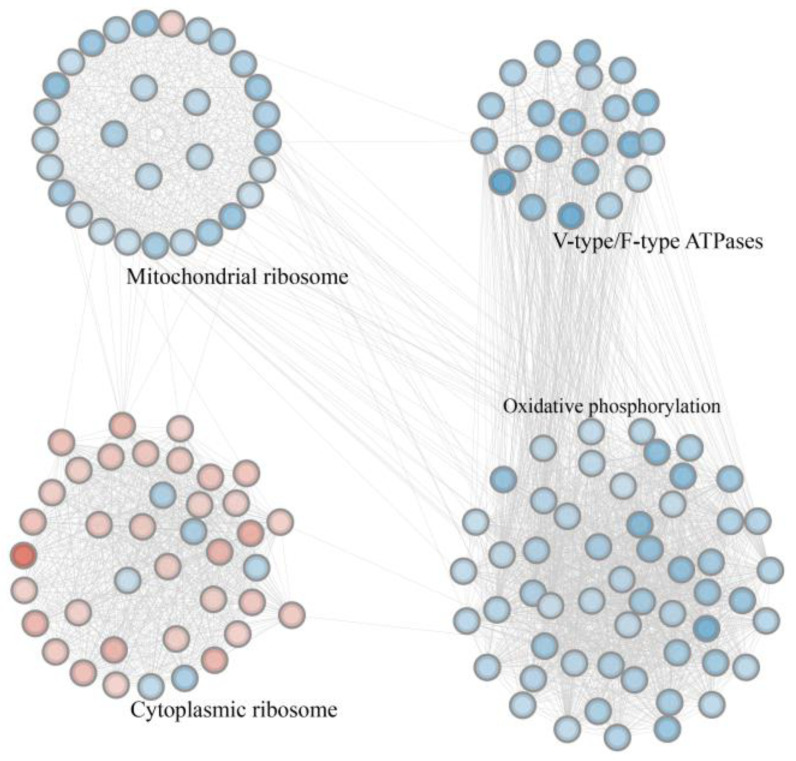
The highest-density modules identified using the protein–protein interaction network. Nodes represent genes, with downregulated genes shown as blue circles and upregulated genes shown as red circles. Black labels denote the primary functions of the genes within each block. The black connecting lines indicate interactions between proteins.

A PPI network was constructed based on the significant DEGs. Cytoscape’s MCODE plugin was used to identify the two highest-density modules, which were then exported as subnetworks for subsequent visualization and functional analysis (Figure 5 and Table S10). These genes were primarily clustered into four functional blocks, including V-type/F-type ATPases, mitochondrial ribosomes, cytoplasmic ribosomes, and oxidative phosphorylation. In the V-type/F-type ATPase block, all 22 genes were uniformly downregulated. Similarly, in the oxidative phosphorylation block, all 54 genes were significantly downregulated and enriched in pathways such as oxidative phosphorylation (ko00190), thermogenesis (ko04714), and retrograde endocannabinoid signaling (ko04723). Within the mitochondrial ribosome block, most genes involved in ribosomal subunit biogenesis were downregulated. In contrast, most genes in the cytoplasmic ribosome block were upregulated.

## 4. Discussion

Accurate annotation of genes expressed in specific tissues would provide a foundation for functional studies. In the present study, a comprehensive reference-guided transcriptomic analysis was performed to gain new insights into the gene expression reprogramming in the *A. pernyi* midgut after ApNPV infection. Using the PacBio long-read sequencing approach, 1850 novel genes were annotated, significantly improving the current genomic annotations of *A. pernyi*. In total, 1664 lncRNAs and 858 TFs were identified, providing a comprehensive view of the regulatory landscape within the midgut. The 2471 AS and 2133 APA events revealed the complexity of post-transcriptional regulation in the tissue. All these cases could not be disclosed in the 2016 study [50] due to its short-read sequencing. As the midgut acts as *A. pernyi*’s first antiviral defense site, the improved annotations of genes expressed in the tissue would enable the critical investigation of (ⅰ) the regulatory mechanisms of gene expression and (ⅱ) the tissue-specific functions of lncRNAs and TFs, thereby elucidating their synergistic roles in immune response modulation. Given the importance of these events in fine-tuning gene expression in eukaryotes, the widespread occurrence of these events in *A. pernyi* suggests a high degree of transcriptomic plasticity in response to pathogenic stimuli.

ApNPV is a double-stranded DNA virus that poses a significant threat to large-scale *A. pernyi* farming. Reanalysis of the transcriptomic data obtained during ApNPV infection, integrating both FL and second-generation transcriptomics, revealed 4796 differentially expressed FL isoforms, including those of important immune- and metabolism-related genes. Moreover, 164 and 171 differentially expressed lncRNAs and TFs, respectively, were detected, indicating that virus-induced stress triggers extensive reprogramming of the gene regulatory networks. PPI analysis revealed the downregulation of genes involved in pathways associated with mitochondrial ribosomes, F-type ATPases, and oxidative phosphorylation, indicating the inhibition of energy metabolism in *A. pernyi*. Conversely, most genes related to cytoplasmic ribosomal proteins were upregulated, suggesting that the virus exploits the host translational machinery to facilitate its own replication. Collectively, these findings indicate that ApNPV infection may lead to the coordinated remodeling of the host’s biosynthetic and metabolic processes, which may be a strategy to reprogram cellular resources and promote viral propagation. Alternatively, it is also possible that these changes are the response of *A. pernyi* to viral infection, which limits viral spread by altering its own biological processes.

*Relish*, a TF of the NF-κB family, is involved in multiple cellular processes, such as cell proliferation, apoptosis, and autophagy, and functions as a crucial component of the IMD signaling pathway [5,62]. *ApRelish* can regulate autophagy [63]. Here, a novel splice isoform of *ApRelish*, ApRelish.2, was found to be upregulated after ApNPV infection (Figure 3B). Although this isoform contains a truncated 5′ regulatory region and a partial deletion of exon 11, all major domains, including the RHD, IPT, ankyrin repeat, and death domains, are retained, except for the deletion of 11 amino acids between the IPT and ankyrin repeat domains (Figure S3). The 5′-untranslated region (UTR) plays an important role in controlling translation initiation efficiency and mRNA stability [64,65]. The 5′-end truncation of ApRelish.2 may affect its translation and response to ApNPV infection. However, the consequences of the deletion of 11 amino acids remain unclear and should be investigated in the future. *BmRelish* also has two isoforms; the encoded proteins differ at the C-terminus, with one containing an ankyrin repeat domain and the other lacking it, resulting in functional differences in the regulation of antimicrobial peptide expression [66]. The two Relish proteins in *B. mori* and *A. pernyi* are functionally different, indicating possible functional divergence of the isoforms *ApRelish* and *BmRelish* during or after species formation, which requires further study.

*SOCS2*, a key cytokine signaling regulator of the SOCS family, is rapidly induced by activated STAT following JAK/STAT signaling to negatively regulate cytokine signaling via feedback loops [67]. In *B. mori*, *SOCS2* plays a role in suppressing the replication and proliferation of BmNPV by impairing the transcription of the core viral genes [68]. In this study, *ApSOCS2* was found to contain five splice isoforms owing to ES events and different transcription start sites. Among these, three isoforms were significantly upregulated after ApNPV infection, including ApSOCS2.2, a previously reported isoform [69]. Among the encoded proteins, only ApSOCS2.1 lacked the SOCS-box domain, whereas the other variants possessed intact SH2 and SOCS-box domains (Figure S4). The SOCS-box domain is essential for recruiting the E3 ubiquitin ligase scaffold Cullin 5, which catalyzes the ubiquitination of phosphorylated signaling intermediates [70,71]. The absence of the SOCS-box domain in ApSOCS2.1 is predicted to abolish its ability to promote the degradation of signaling intermediates. In addition, BmSOCS2S and BmSOCS2L isoforms exhibited distinct responses to BmNPV [72]. ApSOCS2.2 and ApSOCS2.3 shared similar N-terminal structures with BmSOCS2S and BmSOCS2L, respectively, suggesting that these isoforms may have similar immune functions. Previous research has demonstrated a strong negative correlation between the length of a transcript’s 5′UTR and its expression level [73]. The observed variations in the 5′-UTR regions among *ApSOCS2* isoforms may lead to differences in expression levels, thereby indirectly influencing the functional regulation of ApSOCS2.

The genes upregulated in the *A. pernyi* midgut after ApNPV infection were significantly enriched in autophagy and apoptosis pathways. Autophagy functions not only as an antiviral pathway but also as a mechanism that promotes viral proliferation. Although autophagy typically degrades viral particles or components by delivering them to lysosomes [74,75], viruses sometimes evade this process or exploit it to bypass host immune defenses, thereby enhancing their replication and dissemination [76,77]. BmNPV can induce host autophagy to facilitate its replication within cells while concurrently causing mitochondrial injury [78,79]. Two autophagy-related pathways (ko04136 and ko04140) were induced in the *A. pernyi* midgut, indicating enhanced autophagic activity and probable viral reprogramming of the autophagic process to affect ApNPV proliferation. The observed suppression of energy metabolism-related pathways, such as oxidative phosphorylation (ko00190) and thermogenesis (ko04714), may stem from the cascading effects of autophagy-triggered mitochondrial damage. Therefore, these issues must be addressed in future studies. Similarly, during the early stages of viral infection, viruses often inhibit apoptosis to maintain normal viral replication [80]. However, in the later stages, viruses may induce the breakdown of infected cells to facilitate their dissemination [81]. Influenza A and porcine reproductive and respiratory syndrome viruses regulate apoptosis to facilitate their replication and transmission [82,83]. The apoptosis pathway (ko04210) was activated after ApNPV infection, suggesting that changes in apoptosis may affect ApNPV progression in the host.

The upregulated genes in the *A. pernyi* midgut after ApNPV infection were enriched in GO terms such as catalytic activity, acting on DNA (GO:0140097), DNA polymerase activity (GO:0034061), nucleotidyltransferase activity (GO:0016779), and catalytic activity, acting on nucleic acids (GO:0140640); all of these GO terms are associated with DNA synthesis and replication (Table S6). These genes may also be involved in viral replication and midgut cell replenishment. As obligate intracellular parasites, viruses depend entirely on host cell’s resources for replication. Numerous viruses, including H5N1, Kaposi’s sarcoma-associated herpes virus, and Israeli acute paralysis virus, have been reported to hijack host cytoplasmic ribosomes to synthesize essential enzymes and structural capsid proteins critical for their propagation [84,85,86]. Upon infection, many viruses suppress the translation of host mRNAs while selectively enhancing the translation of ribosomal protein mRNAs and promoting ribosome biogenesis [87,88]. Avian infectious bronchitis virus can induce proteomic changes in the host that delay cell cycle progression; however, it also enhances viral translation by promoting ribosomal biogenesis [89,90]. In addition to utilizing ribosomes for protein synthesis, certain ribosomal proteins contribute to viral infections through their extra-ribosomal functions. For example, uS9/RPS16 has been shown to enhance influenza A virus replication by modulating TBK1 phosphorylation and attenuating type I interferon signaling [91]. Here, the cytoplasmic ribosomal subnetwork was activated after ApNPV infection (Figure 5), indicating that the *A. pernyi* translational machinery may be used by ApNPV. These results imply that ApNPV might utilize not only the translational machinery for protein production but also potentially co-opt the DNA replication system to facilitate viral genome replication in *A. pernyi*. Given that viral infection increases midgut cell division in mosquitoes, representing an important aspect of the antiviral response [92], we cannot rule out that the upregulation of DNA synthesis here also suggests induced proliferation of intestinal cells and a host immune response to limit viral proliferation in *A. pernyi*, which requires further elucidation.

Mitochondrial ribosomal proteins are generally encoded by nuclear genes and synthesized in the cytoplasm before being transported to the mitochondria to assemble mitochondrial ribosomes. Mitochondrial ribosomes are specifically responsible for translating mitochondria-encoded proteins essential for oxidative phosphorylation [93,94,95]. Many genes encoding mitochondrial ribosomal proteins were downregulated after ApNPV infection (Figure 5), which may have suppressed the translation of mitochondrial genes and affected oxidative phosphorylation. In addition, the expression of oxidative phosphorylation-related genes was downregulated after ApNPV infection (Figure 5), indicating that oxidative phosphorylation was also inhibited at the transcriptional level. This suppression indicates the impairment of mitochondrial function. The upregulation of cytoplasmic ribosome-related genes suggests viral reprogramming, likely via commandeering of the host’s translational machinery by the virus for its own protein synthesis and proliferation.

V-type ATPases utilize the energy generated from ATP hydrolysis to drive proton transport across intracellular membranes, leading to the acidification of various organelles [96]. F-type ATPases are essential components of the oxidative phosphorylation pathway that drives ATP synthesis and supports energy metabolism [97]. V-type ATPases in *B. mori* are involved in the degradation of BmNPV by mediating the acidification of endosomes and lysosomes [98]. In the *A. pernyi* midgut after ApNPV infection, we observed that genes associated with V-type ATPases were significantly downregulated (Figure 5). This downregulation may impair organelle acidification and correspond to the suppression of activity in the lysosome (ko04142) and phagosome (ko04145) pathways (Table S7). These results suggest that the inhibition of V-ATPase function may impair the antiviral mechanisms in *A. pernyi*. Moreover, the downregulation of F-type ATPase genes was associated with the downregulation of genes enriched in important energy metabolism-related pathways, such as oxidative phosphorylation (ko00190) and thermogenesis (ko04714). Additionally, other metabolism-related pathways, such as valine, leucine, and isoleucine degradation (ko00280), N-glycan biosynthesis (ko00510 and ko00513), fatty acid degradation (ko00071), ubiquinone and other terpenoid–quinone biosynthesis (ko00130), glutathione metabolism (ko00480), and butanoate metabolism (ko00650), were also inhibited. These results indicate that ApNPV may interfere with the energy metabolism of *A. pernyi*, which may be a strategy for viral proliferation. Given that viral proliferation requires host energy supply, downregulation of metabolism during infection may also be a host immune response to infection. Further in-depth studies are necessary.

The evolutionary arms race between viruses and their hosts has driven viruses to evolve various strategies to disrupt host defenses and exploit cellular resources for replication [19]. Our results indicate that, after ApNPV infection, the biogenesis of mitochondrial ribosomes, oxidative phosphorylation, and energy metabolism in *A. pernyi* are suppressed, whereas autophagy, apoptosis, and cytoplasmic ribosome biogenesis are exploited. These findings suggest that the virus may reprogram *A. pernyi* midgut cells to achieve rapid proliferation and spread by suppressing energy metabolism and immune functions. Further studies are required to elucidate how ApNPV exploits these cellular functions for immune evasion and proliferation.

## 5. Conclusions

In this study, an FL transcriptome atlas of the *A. pernyi* midgut was constructed to systematically improve the annotations of the genes expressed in the tissue, including many immune- and metabolism-related genes and their novel isoforms. By reanalyzing the existing RNA-seq data following ApNPV infection, we found that the virus reprograms critical functional modules within *A. pernyi* midgut during invasion. These include the pathways governing cell autophagy and apoptosis, as well as the key cellular processes: V-/F-type ATPase activity, mitochondrial and cytosolic ribosome functions, and oxidative phosphorylation. These findings indicate that ApNPV may promote its own inter-tissue transmission by activating midgut cell autophagy and apoptosis pathways during invasion. Concurrently, the virus may suppress the mitochondrial ribosome function, oxidative phosphorylation, and V-/F-type ATPase activity to disrupt the energy production and organelle acidification within the midgut. During these processes, the host translational system may be also exploited. In addition, differentially expressed lncRNAs and TFs may play a potential regulatory role during viral infection. In summary, our study provides preliminary evidence that ApNPV may achieve efficient proliferation and inter-tissue transmission by reprogramming midgut energy metabolism, macromolecular synthesis, and immune processes. Future studies should focus on elucidating the precise mechanisms of host-pathogen interactions, particularly the viral strategies employed to subvert midgut defenses for successful viral proliferation and dissemination.

## Figures and Tables

**Table 1:** Summary of PacBio Iso-Seq data from the FL transcriptome of the *A. pernyi* midgut.

	**Total Bases (Gbp)**	**Total Number**	**Minimum Length (bp)**	**Average Length (bp)**	**Maximum Length (bp)**	**N50 (bp)**
Raw reads	39.48	550,771	57	71,679	358,048	128,749
Subreads	38.01	19,093,122	51	1991	223,063	2269
CCS	0.79	378,156	109	2081	14,486	2321
FLNC with ploy(A)	0.55	288,759	57	1888	9624	2113

N50 represents the minimum read length required to cover 50% of the total length when reads are sorted by descending length.

## Data Availability

The FL raw data have been deposited in the National Genomics Data Center (NGDC), China National Center for Bioinformation (CNCB)/Beijing Institute of Genomics, Chinese Academy of Sciences, under the accession number CRA025977, and are publicly accessible on 24 May 2025 at https://ngdc.cncb.ac.cn/gsa/.

## Supplementary Materials

The following supporting information can be downloaded at https://www.mdpi.com/article/10.3390/insects16080792/s1, Table S1: Functional annotation of the novel protein-coding genes expressed in the midgut of *A. pernyi.* Five public databases, namely, NCBI non-redundant protein sequence (NR), Gene Ontology Consortium (GO), Kyoto Encyclopedia of Genes and Genomes (KEGG), Eukaryotic Orthologous Groups (KOG), and Swiss-Prot databases, were used for gene annotation. KO represents KEGG Orthology. The gene IDs and their functional annotations based on public databases are listed. A dash (“–”) indicates the lack of annotation information in the corresponding database; Table S2: Transcription factors identified in the midgut of *A. pernyi.* The correspondence between TF genes and TF families is shown in Sheet1. The corresponding relationship between the TF families and their characteristic domains is shown in Sheet2. The gene IDs were used to retrieve their sequences from the *A. pernyi* genome [32]; Table S3: Classification and statistics of alternative splicing events. Each row shows each detected splicing event (Column D) in one gene (Column A), including the involved isoform (Column B), indicated structural classification (Column C), and splice-junction coordinate (Column E); AA, AD, ES, MEE, and IR represent the alternative splice events of alternative acceptor, alternative donor, exon-skipping, mutually exclusive exon, and intron retention, respectively. Table S4: Summary of APA events discovered in the midgut of *A. pernyi*. The number and position of APA sites in each gene (Column A) are shown in Columns D and E, respectively. Aligned reads represent the support reads for each APA event, and Other Columns show the function annotations of the gene in five public databases: NR, GO, KEGG, KOG, and Swiss-Prot; Table S5: Differential expression of isoforms in canonical immune pathways. The expression values for each isoform in the Ap_CK and Ap_NPV groups are shown. The *p* value indicates the significance of each differentially expressed isoform. FDR and logFC are abbreviations for false discovery rate and log_2_(FoldChange), respectively. LogFC quantifies the direction and magnitude of expression changes, with significant changes typically determined by combining logFC with FDR. Up indicates the upregulated expression. Down represents the downregulated expression. No indicates that the isoform expression was unchanged. Functional annotations of isoforms in the five public databases: NR, GO, KEGG, KOG, and Swiss-Prot, are shown; Table S6: GO enrichment analysis of differentially expressed genes. Up- and downregulated genes were enriched, respectively. GO terms and their corresponding IDs are shown. The numbers and IDs of all genes enriched in each GO term are listed. The *p* value indicates the significance of the enriched GO term, and the Q value represents the adjusted *p* value. Column H (Gene ID) shows all the genes enriched for a specific GO term. The gene number represents the number of genes enriched for a specific GO term; Table S7: KEGG enrichment analysis of the differentially expressed genes. Up and Down show the upregulated and downregulated genes enriched, respectively. The KEGG pathway and its corresponding IDs are shown. The numbers and IDs of all genes enriched in each pathway are listed in Columns F and I, respectively. The *p* value indicates the significance of each enriched pathway, and the Q value represents the adjusted *p* value; Table S8: Statistics of differentially expressed lncRNAs in the *A. pernyi* midgut after ApNPV infection. Transcript ID represents the ID of differentially expressed lncRNAs. The expression values for each lncRNA in the Ap_CK and Ap_NPV groups are shown. The *p* value indicates the significance of each differentially expressed lncRNA. FDR and logFC were also used together to determine the differential expression of each lncRNA. Up, Down, and No suggest the upregulated, downregulated, and unchanged expression, respectively; Table S9: Differentially expressed TFs in the *A. pernyi* midgut after ApNPV infection. Columns A and B represent the ID of the TF gene and its corresponding TF family, respectively. The expression values for each TF gene in the Ap_CK and Ap_NPV groups are also shown. The *p* value indicates the significance of each differentially expressed TF gene. FDR and logFC were also used together to determine the differential expression of each TF gene. The upregulated, downregulated, and unchanged expression of TF genes are shown by Up, Down, and No, respectively; Table S10: Top highest-density modules from the protein–protein interaction network. Four functionally similar blocks are shown in Column A (Group). The scores represent the cluster scores of functionally relevant genes in the module. Node status indicates each gene’s position in a module, clustered or seed, where cluster denotes the belonging relationship of each gene to a module. The details of every DEG in these blocks are presented, including the Gene ID, functional annotations in five public databases (NR, GO, KEGG, KOG, and Swiss-Prot), and the expression values in the Ap_CK and Ap_NPV groups. Up, Down, and No are also used to show the upregulated, downregulated, and unchanged expression of each gene, respectively; Figure S1: GO categorization of novel protein-coding genes. Different colors represent the three main ontologies: biological process, molecular function, and cellular component. Each outermost circle indicates one GO term. The circle size is scaled with the count of enriched genes; Figure S2: Heatmap showing the differential expression of isoforms in canonical immune pathways after ApNPV infection. The expression values were transformed to log_2_(FPKM + 1) to normalize the variance. Row-wise Z-score normalization was applied (mean = 0, SD = 1), enabling cross-sample comparison of relative upregulation/downregulation. The color key indicates the expression level; Figure S3: Multiple sequence alignment and conserved domain analysis of the ApRelish proteins. Differentially colored underlines show the four conserved domains: RHD, IPT, Ankyrin, and Death. Dashes indicate the deficiency of 11 amino acids in ApRelish.2 due to the choice of the splice site in exon 13; Figure S4: Multiple sequence alignment and conserved domain analysis of the ApSOCS2 and BmSOCS2 proteins. The important domains of SH2 and SOCS-Box are highlighted using red and green underlines, respectively. Dashes represent the gaps introduced to optimize the alignment.
