# Supplementary figures and images for: Integrating Full-Length and Second-Generation Transcriptomes to Elucidate the ApNPV-Induced Transcriptional Reprogramming in Antheraea pernyi Midgut
Source: Insects. 2025 Jul 31;16(8):792. doi: 10.3390/insects16080792 (PMC12386421; doi:10.3390/insects16080792)

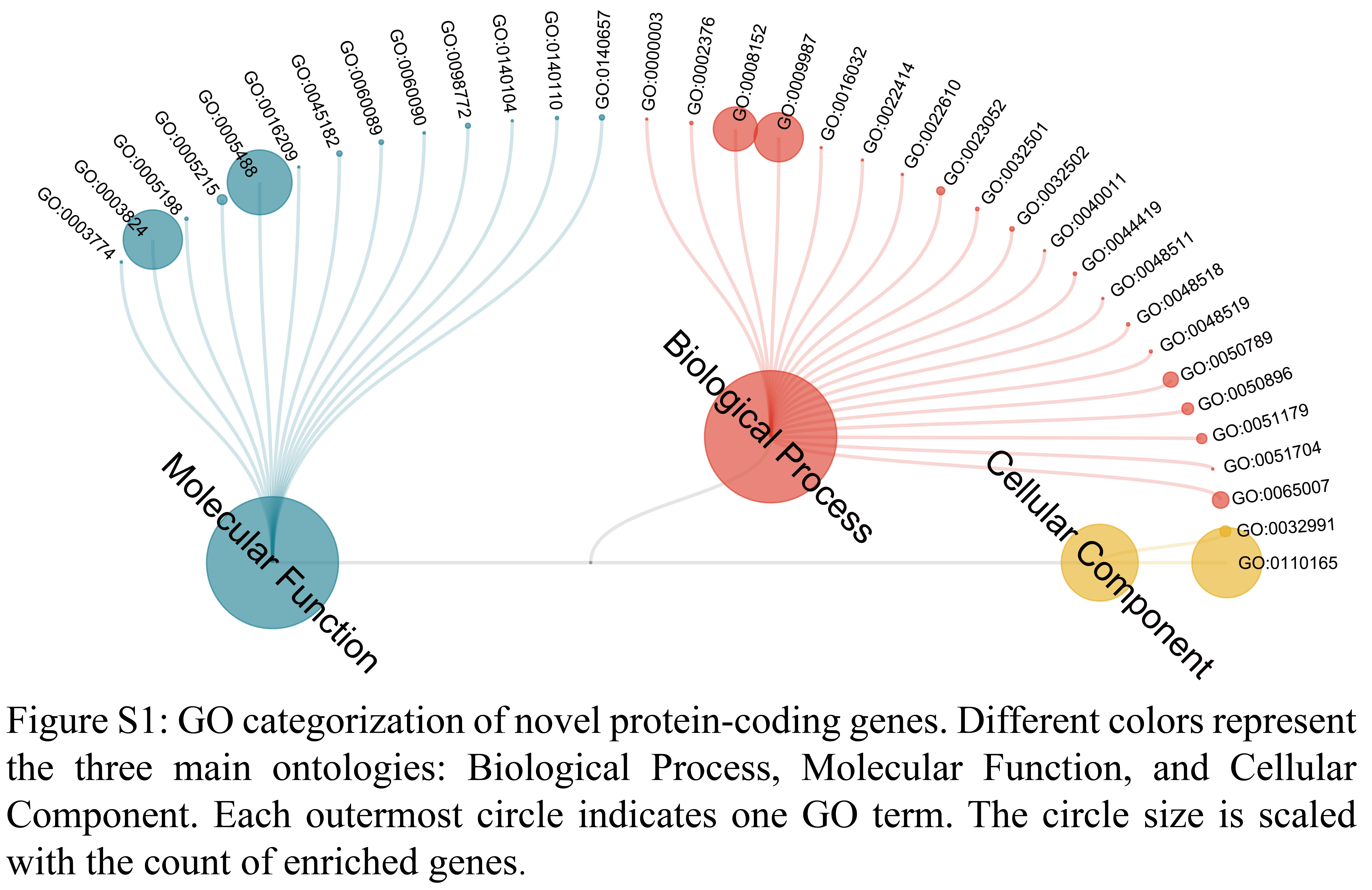

Supplement: Supplementary file 1 [file insects-16-00792-s001.zip › Figure S1.tif]

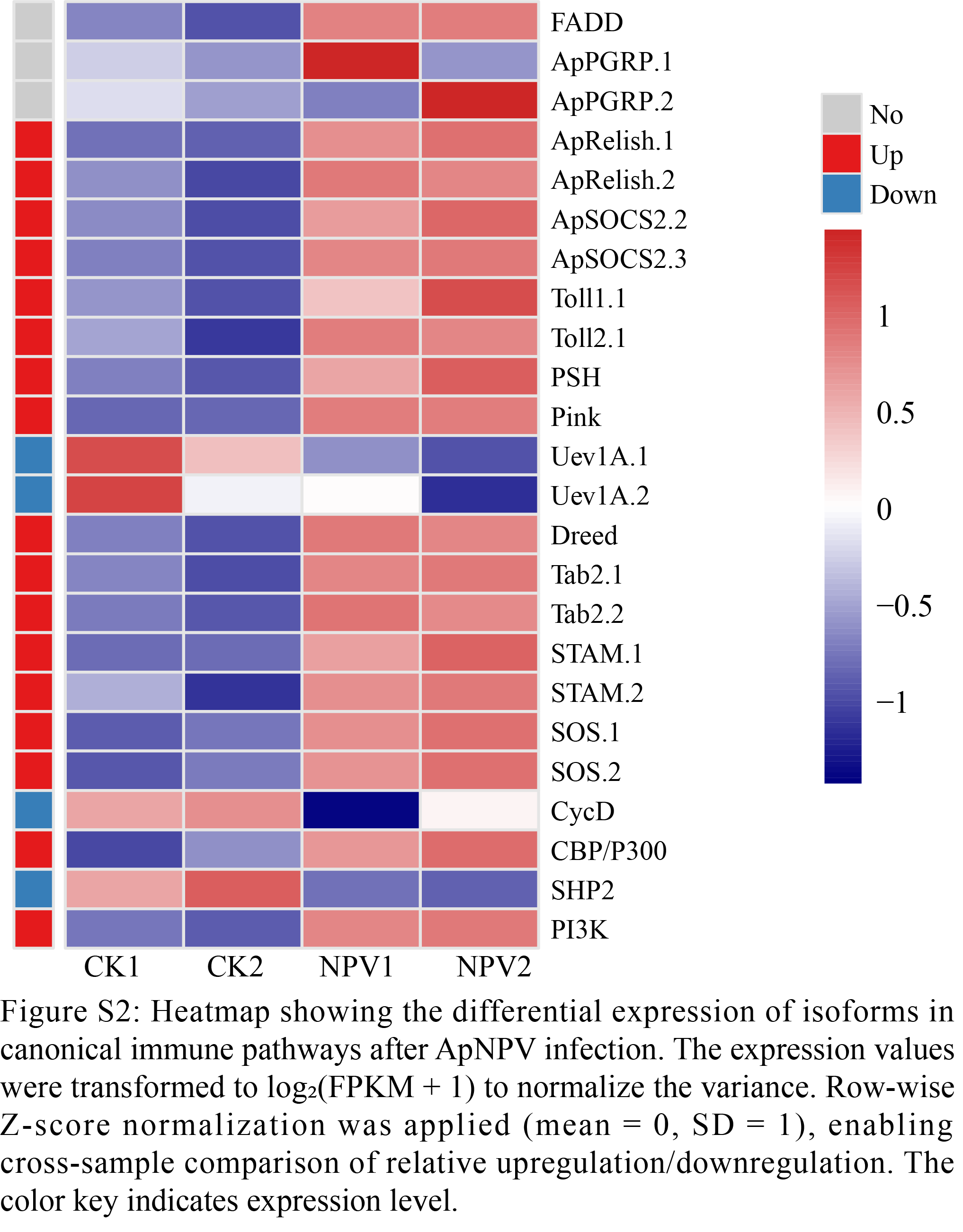

Supplement: Supplementary file 1 [file insects-16-00792-s001.zip › Figure S2.tif]

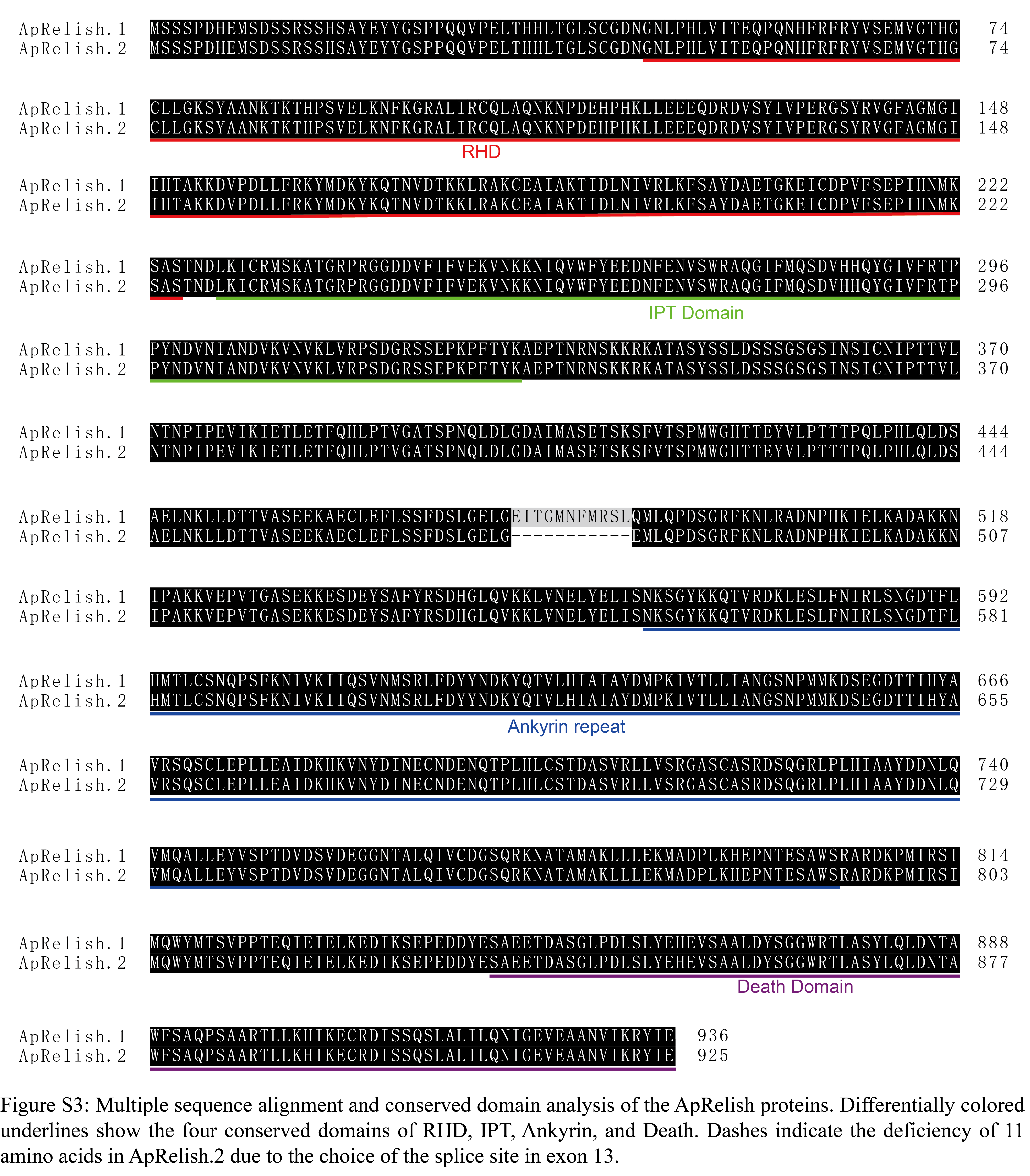

Supplement: Supplementary file 1 [file insects-16-00792-s001.zip › Figure S3.tif]

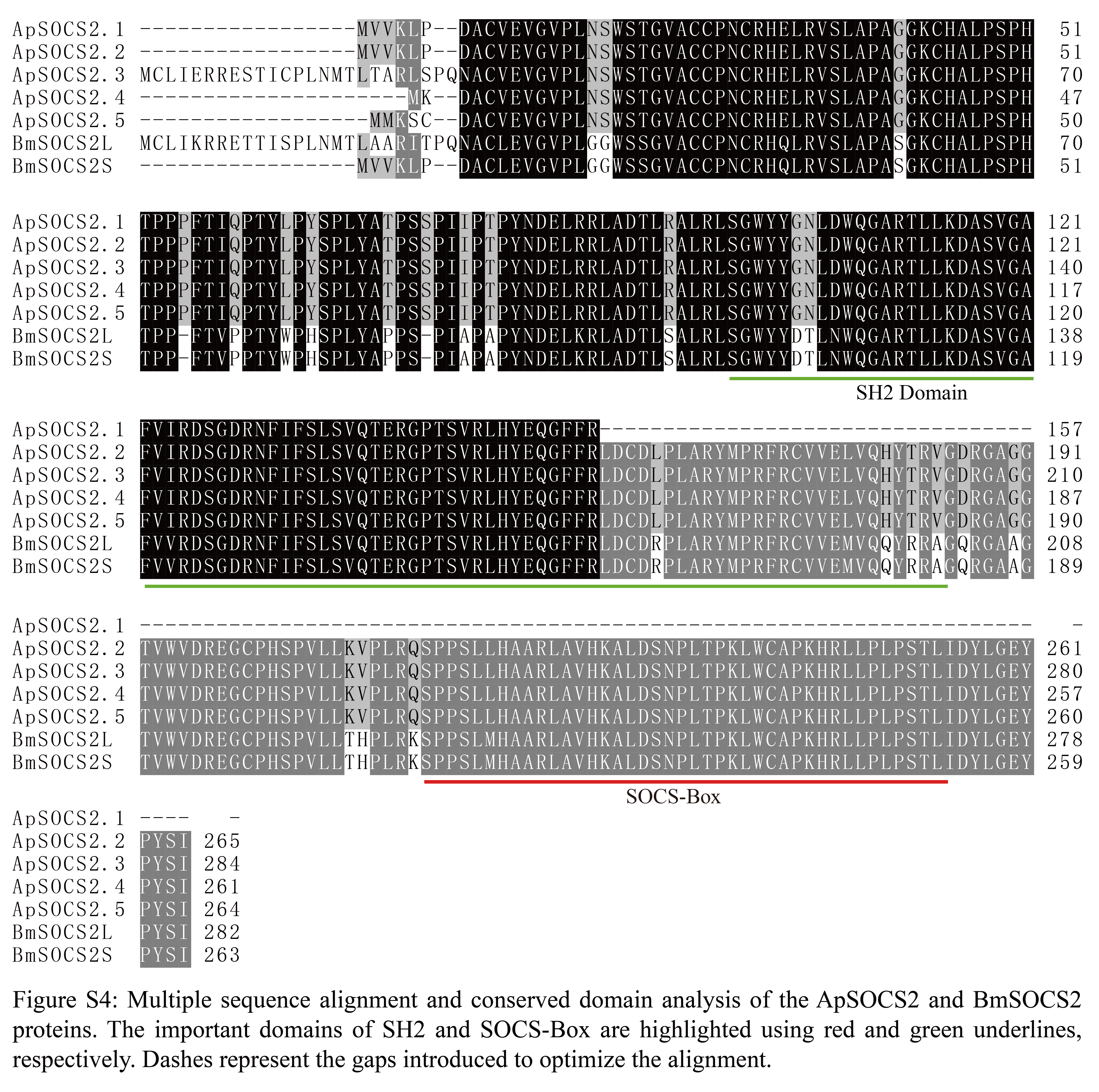

Supplement: Supplementary file 1 [file insects-16-00792-s001.zip › Figure S4.tif]
